# Passive Smartphone Sensing Reveals Gait and Typing Biomarkers of Cognitive Impairment

**DOI:** 10.1002/alz70856_107368

**Published:** 2026-01-09

**Authors:** Yufei Shen, Jared F Benge, Justin Rousseau, Rosemary A Lester‐Smith, Edison Thomaz

**Affiliations:** ^1^ The University of Texas at Austin, Austin, TX, USA; ^2^ UT Southwestern Medical Center, Dallas, TX, USA

## Abstract

**Background:**

Early detection and continuous monitoring of Alzheimer's disease and related dementias are critical for implementing treatments and determining the efficacy of interventions. However, traditional clinical assessments face challenges such as being time consuming, relatively infrequent administration, and insensitivity to the earliest biological changes of the disease. In contrast, passive smartphone sensing presents a promising alternative by leveraging everyday device interactions to unobtrusively monitor a host of functions in real‐world environments over longer periods of time. The TechSANS study (techsans.ece.utexas.edu) aims to discover digital biomarkers from passive smartphone sensing to support naturalistic and scalable functional assessment beyond the clinic.

**Method:**

21 older adults aged ≥ 65 were recruited from the Austin Metropolitan Area. Participants were enrolled for one year and underwent cognitive assessments every 6 months. Passive multimodal smartphone sensor data was continuously collected from an iOS application installed. Measures captured included motion type inferences, gait parameters, location tracking, phone usage, keyboard typing, and communication events. These digital measures were extracted from daily sensing data during the first 6 months of each participant's enrollment and were analyzed against the baseline cognitive categorization (17 cognitively normal, 4 with MCI/dementia) to identify potential digital biomarkers that separate the groups.

**Result:**

Generalized linear mixed models, controlled for age, found the binary cognitive categorization as a significant predictor for gait and typing measures after false discovery rate correction. Specifically, MCI/dementia diagnoses are associated with greater variations in the hold time of character keys during everyday typing tasks (*p* = .03) and greater walking asymmetry (*p* = .04), defined as the proportion of steps taken at different speeds between the left and right foot.

**Conclusion:**

Preliminary statistical analyses from an ongoing study identified gait metrics and keyboard typing patterns captured through passive smartphone sensing as digital biomarkers of cognitive impairment. The findings warrant further research into the relationship between motor performance and cognitive impairment, as well as the development of computational models for the cross‐sectional and longitudinal prediction and characterization of cognitive impairment.

